# Repeat stereotactic radiofrequency thermocoagulation in patients with hypothalamic hamartoma and seizure recurrence

**DOI:** 10.1002/epi4.12378

**Published:** 2020-01-18

**Authors:** Hiroshi Shirozu, Hiroshi Masuda, Shigeki Kameyama

**Affiliations:** ^1^ Department of Functional Neurosurgery National Hospital Organization Nishiniigata Chuo Hospital Niigata Japan; ^2^ Hypothalamic Hamartoma Center National Hospital Organization Nishiniigata Chuo Hospital Niigata Japan; ^3^ Department of Neurosurgery Saiseikai Niigata Hospital Niigata Japan

**Keywords:** gelastic seizure, hypothalamic hamartoma, recurrence, reoperation, stereotactic radiofrequency thermocoagulation

## Abstract

**Objective:**

To evaluate the feasibility of repeat stereotactic radiofrequency thermocoagulation (re‐SRT) for patients with hypothalamic hamartoma (HH) and to clarify clinical and surgical factors for seizure outcomes.

**Methods:**

Hypothalamic hamartoma patients with gelastic seizures (GSs) who underwent SRT were retrospectively reviewed. Seizure outcomes were evaluated separately for GS and other types of seizures (non‐GS). Surgical complications were compared between re‐SRT and first SRT. Clinical and surgical factors related to both seizure recurrences after first SRT and final seizure outcomes were analyzed.

**Results:**

Participants comprised 150 patients (92 males; median age at surgery, 8 years; range, 1.7‐50 years). Of those, 122 (81.3%) had non‐GS. Forty‐three patients (28.7%) underwent re‐SRT. Freedom from GS was achieved by first SRT in 103 patients (68.7%), second SRT in 30/40 (67.5%), third SRT in 3/10 (30.0%), and fourth SRT in 2/3 (66.7%). Finally, 135 patients (90.0%) became GS‐free. Ninety patients (73.8%) achieved non‐GS freedom, with first SRT in all except one case. Transient complications were more frequent with first SRT (118/150, 78.7%) than re‐SRT (35/56, 62.5%), whereas persistent complications were more frequent with re‐SRT (7/56, 12.5%) than with first SRT (3/150, 2.0%). Multivariate analyses revealed only younger age at surgery (≤1 year) as related to GS recurrence after first SRT, with no variables affecting final GS outcomes. Meanwhile, seizure type (tonic seizure), intellectual disability, and genetic syndromes were significant factors for both non‐GS recurrence and final outcomes. Multiple previous treatments were significantly related to final non‐GS outcomes as well. Size and subtype of HH and surgical factors were unrelated to seizure outcomes.

**Significance:**

Repeat stereotactic radiofrequency thermocoagulation provides potential opportunities to achieve freedom from recurrent GS, albeit with increased risks of persistent complications. Non‐GS and intellectual disability could offer early surgical indications, and repeated ineffective treatments should be avoided.

**Key Points**

- Repeat stereotactic radiofrequency thermocoagulation (re‐SRT) was effective for recurrent gelastic seizures (GSs) with tolerable safety
- Re‐SRT was ineffective for recurrence of other types of seizure (non‐GS)
- Younger age at GS onset correlated with GS recurrences but not with final GS outcomes, so SRT can eliminate GS even in older patients
- Tonic seizure, intellectual disability, genetic syndromes, and multiple previous treatments correlated significantly with non‐GS outcomes
- Early indication for SRT would improve seizure outcomes in patients with non‐GS and intellectual disability

## 1 INTRODUCTION

Hypothalamic hamartoma (HH) causes a unique syndrome comprising drug‐resistant gelastic seizures (GSs) as well as multiple types of seizure and intellectual/behavioral problems.^1^ HH is known to have an intrinsic epileptogenesis,^2^, ^3^, ^4^, ^5^ and treatment for HH itself provides not only a chance of freedom from seizures, but also improvement of intellectual/behavioral problems.^6^, ^7^, ^8^, ^9^, ^10^ Although various treatments have been attempted, deep‐seated HH is difficult to approach and satisfactory seizure outcomes have not been obtained. One reason is considered to be that every treatment shows limitations in the size, shape, and location of the HH that can be addressed.^11^, ^12^, ^13^ Combinations of various treatments or staged surgeries are thus recommended, especially for HH with a complex shape or large size.^14^ Repeat surgery is also considered for selected patients with HH in whom the initial surgery failed.^15^, ^16^ Reoperation may provide an additional opportunity for favorable seizure outcomes, but is also associated with additional invasiveness and complications specific to each treatment procedure.

We have been performing only stereotactic radiofrequency thermocoagulation (SRT) for HH treatment since 1997^5^, ^17^ and have reported excellent seizure outcomes from 100 patients with HH.^9^ In that report, repeat SRT (re‐SRT) proved effective against recurrence of GS in 32 patients. We therefore consider SRT as the single option available for the treatment of HH of every size, shape, or location, and even in cases with previous treatment. However, re‐SRT could not achieve freedom from residual/recurrent non‐gelastic seizures (non‐GS). The clinical factors related to the recurrence of GS and non‐GS remain unclear.

The present study aimed to evaluate the effectiveness and feasibility of re‐SRT, and furthermore, to analyze clinical factors related to seizure outcomes after SRT. This investigation would help to develop a definite strategy for HH treatment, including optimal timing of surgery.

## 2 METHODS

### 2.1 Patients

Patients with HH who had GS and underwent SRT at Nishiniigata Chuo Hospital between 1997 and 2015 were retrospectively reviewed. Postoperative seizure and functional outcomes were followed up for more than 1 year after last SRT.

Clinical factors such as age, sex, HH classification and size, seizure type and frequency, behavioral disorders (BD), intellectual disability (ID), precocious puberty (PP), and previous treatments were evaluated. Surgical factors including numbers of trajectories and coagulations were also evaluated. HH subtypes were classified into four categories according to the classification provided in a previous study^17^: parahypothalamic type (P); intrahypothalamic type (I); mixed type with unilateral attachment (MU); and mixed type with bilateral attachment (MB). Size of the HH was evaluated by the maximum diameter in any dimension. Seizure frequency was categorized as daily or non‐daily. BD was judged by the presence of violence, aggressiveness, irritability, or lack of concentration. Autistic symptoms were not included in BD in this study. ID was defined as intellectual quotient or developmental quotient <70 as evaluated by neuropsychotherapists. Most cases of PP had been diagnosed by referring doctors, while some patients presented with PP after SRTs (defined as “delayed PP”). Details of other preoperative evaluations including neuroimaging and the procedure for SRT have been described elsewhere.^9^, ^17^, ^18^ Regular follow‐up was performed at the 3‐month visit and at annual visits up to 5 years after SRT. Surgical complications were classified into three categories: transient, prolonged, or persistent. The term “transient” was used for complications that improved within 2 weeks, and “prolonged” for those healed by the 1 year postoperative visit. Seizure outcomes were divided into seizure‐free or otherwise, and were evaluated separately for GS and non‐GS.

### 2.2 Indications and procedures for re‐SRT

Patients who had both recurrent GS and residual HH attachment became candidates for re‐SRT. Recurrent GS was defined as GS appearing the same as preoperative GS persisting or recurring after SRT. Patients with only an aura or ambiguous preictal feeling were not considered as indicated for reoperation. The indication of re‐SRT was determined at least 3 months after SRT, because some patients who showed recurrent GS immediately after SRT later showed reduced or eliminated seizures. Follow‐up MRI to make this decision was also performed at least 3 months after SRT. Because MRI performed within 1 month after SRT showed swelling or edema, the residual part of the HH was not precisely determined. If a patient with recurrent GS did not show any apparent residual part of the HH that could be the target for re‐SRT, they were not considered a candidate for reoperation. Patients with recurrent non‐GS were not usually indicated for reoperation, unless the patient or their family was eager for performance of re‐SRT.

The procedure for re‐SRT is broadly the same as that for the initial SRT. Burr holes are usually made at different sites from the previous SRT, because the brain surface cannot be precisely recognized due to tissue adhesion at the previous burr hole. Attempts are made to keep burr holes within the same skin incision, but elongation of the skin incision is sometimes necessary. Although re‐SRT was performed using an approach on the side contralateral to the previous surgery for several patients until 2014, re‐SRT was set to use an approach from the side ipsilateral to the previous surgery from mid‐2014.

### 2.3 Comparison and statistical analyses

Clinical factors, surgical procedures, and outcomes were compared among HH subtypes. Surgical procedures and complications were compared between first SRT and re‐SRT. Clinical and surgical factors related to recurrence after first SRT and final seizure outcomes were evaluated for GS and non‐GS, separately.

Continuous variables are expressed as median, range, and interquartile range (IQR). Categorical variables are summarized as the number of patients and percentages.

Nominal and continuous variables for comparison among HH subtypes were analyzed using Pearson's chi‐square test and the Kruskal‐Wallis test, respectively. Univariate analyses of clinical factors for seizure recurrences and final seizure outcomes were performed using the chi‐square test. Continuous variables were dichotomized about the median. Multivariate logistic regression was used to calculate odds ratios (ORs) and 95% confidence intervals (95% CIs) after controlling simultaneously for potential confounders. Variables considered in the models were age at surgery and GS and non‐GS onset, duration of GS and non‐GS, BD, ID, genetic syndromes, and previous treatments (open surgery, gamma knife radiosurgery [GKS], and multiple treatments). In addition, seizure types (complex partial seizure, tonic seizure, and generalized tonic‐clonic seizure) were used for analysis of non‐GS outcomes.

Statistical analysis was performed using JMP^®^ version 13.0 (SAS Institute). ORs and 95% CIs were calculated with values of *P* < .05 considered statistically significant.

This retrospective study was approved by the ethics board at our institute (No. 1830).

## 3 RESULTS

### 3.1 Patient profiles

A total of 150 patients (92 males and 58 females) were enrolled. Some overlap in the study population (99 of 150 patients) was seen between this and a previous investigation.^9^ Patient profiles are provided in Table 1. All patients had GS, and 122 patients (81.3%) also had non‐GS. Only six patients (4.9%) presented with non‐GS first, followed by GS. Median maximum diameter of HH was 15 mm (range, 4.5‐80 mm; IQR, 10‐31 mm).

**Table 1:** Patient profiles

	Overall	Parahypothalamic type	Intrahypothalamic type	Mixed type		*P* value
				Unilateral attachment	Bilateral attachment	
Number	150	8 (5.3%)	35 (23.3%)	39 (26.0%)	68 (45.3%)	
Sex, male (%)	92 (61.3%)	3 (37.5%)	19 (54.3%)	28 (71.8%)	42 (61.8%)	.22
Age at surgery, y	8 (1.7‐50, 4‐16)	15 (3‐48, 3.75‐30)	13 (2‐50, 7‐28)	10 (2‐38, 6‐29)	5 (1.7‐42, 3‐12.5)	<.001
Age at GS onset, y	0.8 (0‐11, 0‐2)	1.15 (0‐10, 0.13‐7.25)	1 (0‐11, 0.2‐2)	1 (0‐6, 0‐3)	0.4 (0‐10, 0‐1)	.13
Duration of GS, y	6 (0‐47, 3‐14.63)	10.5 (2.2‐38, 2.88‐28.88)	12 (1‐47, 5‐27)	6.5 (1‐32, 3.5‐17)	4 (0‐41.5, 2‐9.75)	<.001
Age at non‐GS onset, y	4 (0‐28, 1.5‐9)	5 (0‐20, 3‐11)	6 (0.3‐28, 3‐9.75)	5.5 (0‐15, 2‐10)	2.2 (0‐19, 0.5‐4.75)	<.001
Duration of non‐GS, y	4.35 (0‐45, 1.35‐11.45)	18 (2‐28, 5.75‐27.25)	6 (0‐45, 2‐18)	4.6 (0‐26, 1.25‐11.15)	3 (0‐38.5, 1‐9.75)	.03
Maximum diameter, mm	15 (4.5‐80, 10‐31)	11.5 (4.5‐18, 10‐14.5)	10 (5‐15, 8‐10)	14 (7‐35, 11‐17)	22 (9‐80, 18‐30)	<.001
GS frequency						
Daily	134 (89.3%)	7 (87.5%)	33 (94.3%)	34 (87.2%)	60 (88.2%)	.77
Non‐daily	15 (10.0%)	1 (12.5%)	2 (5.7%)	5 (12.8%)	7 (10.3%)	
Unknown	1 (0.7%)				1 (1.5%)	
Non‐GS (+)	122 (81.3%)	6 (75.0%)	28 (80.0%)	36 (92.3%)	52 (76.5%)	.22
Preceding non‐GS	6 (4.9%)					
CPS	76 (50.7%)	3 (37.5%)	23 (65.7%)	25 (32.9%)	25 (36.8%)	.008
GTCS	61 (40.7%)	4 (50.0%)	15 (42.9%)	16 (41.0%)	26 (38.2%)	.91
Tonic Sz	50 (33.3%)	6 (75.0%)	8 (22.9%)	12 (30.8%)	24 (35.3%)	.04
Atonic Sz	15 (10.0%)	1 (12.5%)	1 (2.9%)	4 (10.3%)	9 (13.2%)	.42
Myoclonic Sz	5 (3.3%)	1 (12.5%)	1 (2.9%)	1 (2.6%)	2 (2.9%)	.53
Epileptic spasms	5 (3.3%)	0	1 (2.9%)	3 (7.7%)	1 (1.5%)	.34
Non‐GS frequency						
Daily	44 (36.1%)	3 (50.0%)	7 (25.0%)	12 (33.3%)	22 (42.3%)	.36
Non‐daily	77 (63.1%)	3 (50.0%)	21 (75.0%)	24 (66.7%)	29 (55.8%)	
Unknown	1 (0.8%)				1 (1.9%)	
Behavioral disorder	78 (52.0%)	4 (50.0%)	9 (25.7%)	20 (51.3%)	45 (66.2%)	.002
Intellectual disability	73 (48.7%)	3 (37.5%)	11 (31.4%)	18 (46.2%)	41 (60.3%)	.04
Precocious puberty	49 (32.7%)	5 (62.5%)	1 (2.9%)	8 (20.5%)	35 (51.5%)	<.001
Delayed PP	19 (12.7%)	0	1 (2.9%)	8 (20.5%)	10 (14.7%)	.09
Genetic syndrome	11 (7.3%)	0	0	0	11 (16.2%)	.003
Previous treatments	41 (27.3%)	2 (25.0%)	6 (17.1%)	12 (30.8%)	21 (30.9%)	.47
Open surgery	22 (14.7%)	2 (25.0%)	3 (8.6%)	8 (20.5%)	9 (13.2%)	.41
Gamma knife surgery	22 (14.7%)	1 (12.5%)	4 (11.4%)	5 (12.8%)	12 (17.7%)	.82
Endoscopic surgery	3 (2.0%)	0 (0%)	0 (0%)	0 (0%)	3 (4.4%)	.30
Others	5 (3.3%)	0 (0%)	0 (0%)	2 (5.1%)	3 (4.4%)	.55
Multiple treatments	8 (5.3%)	1 (12.5%)	1 (2.9%)	2 (5.1%)	4 (5.9%)	.73
1st SRT	150	8	35	39	68	
Burr holes	2 (1‐3, 1‐2)	1 (1‐2, 1‐1.75)	1 (1‐2, 1‐1)	1 (1‐3, 1‐2)	2 (1‐3, 2‐2)	<.001
Trajectories	4 (1‐11, 3‐6)	2.5 (1‐4, 1.25‐4)	3 (1‐7, 2‐4)	4 (2‐8, 3‐5)	6 (2‐11, 4‐7)	<.001
Coagulations	8 (1‐36, 5‐14)	3.5 (2‐12, 2‐7.25)	4 (1‐13, 3‐5)	6 (2‐20, 5‐10)	13 (3‐36, 9‐19)	<.001
Re‐SRT	56	2	17	7	30	
Burr holes	1 (1‐3, 1‐2)	1 (1‐1, 1‐1)	1 (1‐1, 1‐1)	1 (1‐1, 1‐1)	1.5 (1‐3, 1‐2)	<.001
Trajectories	3 (1‐7, 2‐4)	2.5 (2‐3, 2‐3)	2 (2‐3, 2‐3)	2 (1‐4, 2‐4)	4 (1‐7, 2‐5)	.01
Coagulations	4 (2‐32, 3‐7.75)	4.5 (3‐6, 3‐6)	3 (2‐6, 2‐4)	2 (2‐8, 2‐6)	6.5 (2‐32, 4‐11.75)	<.001
Final Sz freedom						
Overall Sz	110 (73.3%)	6 (75.0%)	27 (77.1%)	28 (71.8%)	49 (72.1%)	.94
GS	135 (90.0%)	8 (100%)	32 (91.4%)	35 (89.7%)	60 (88.2%)	.75
Non‐GS	90/122 (73.8%)	4/6 (66.7%)	22/28 (78.6%)	27/36 (75.0%)	37/52 (69.2%)	.87
Reoperation	43 (28.7%)	2 (25.0%)	14 (40.0%)	6 (15.4%)	21 (30.9%)	.12
1 SRT	107 (71.3%)	6 (75.0%)	21 (60.0%)	33 (84.6%)	47 (69.2%)	.34
2 SRTs	33 (22.0%)	2 (25.0%)	11 (31.4%)	5 (12.8%)	15 (22.1%)	
3 SRTs	7 (4.7%)	0 (0%)	3 (8.6%)	1 (2.6%)	3 (4.4%)	
4 SRTs	3 (2.0%)	0 (0%)	0 (0%)	0 (0%)	3 (4.4%)	

Note

Binary variables are presented as n (column %). Continuous variables are expressed as median (range, IQR).

Abbreviations: CPS, complex partial seizure; F, female; GS, gelastic seizure; GTCS, generalized tonic‐clonic seizure; IQR, interquartile range; M, male; non‐GS, other types of seizure; PP, precocious puberty; Re‐SRT, repeat SRT; SRT, stereotactic radiofrequency thermocoagulation; Sz, seizure.

The most frequent HH subtype was MB type (n = 68, 45.3%), showing significantly lower median age at both first SRT (5 years; *P* < .001) and non‐GS onset (2.2 years; *P* < .001), and shorter median duration of GS (4 years; *P* < .001) compared with other subtypes. Age at GS onset did not differ among subtypes. Maximum size of HH was significantly greater for MB type (median, 22 mm) than for other subtypes (*P* < .001).

Seizure frequencies of GS and non‐GS did not differ among HH subtypes. Complex partial seizures were most often found in patients with I type (*P* = .008), and tonic seizures were most frequent in patients with P type (*P* = .04).

Patients with I type less frequently showed concomitant BD (9/35, 25.7%; *P* = .002), while those with MB type had a higher incidence of ID (41/68, 60.3%; *P* = .04) compared with other subtypes. PP was seen more frequently in patients with MB (35/68, 51.5%) and P (5/8, 62.5%) types compared with other types (*P* < .001).

Genetic syndromes were recognized in 11 patients (7.3%), all of whom showed MB types. Genetic syndromes included Pallister‐Hall syndrome (n = 8), oral‐facial‐digital syndrome (OFD) type VI (n = 2), and OFD type I (n = 1). All of these were diagnosed by clinical manifestations, and genetic mutations were confirmed in eight patients.

Forty‐one patients (27.3%) had undergone failed previous treatments at other institutes, comprising open surgery (pterional approach or interhemispheric approach) in 22 (14.7%), GKS in 22 patients (14.7%), endoscopic surgery in three (2.0%), and others (vagus nerve stimulation in 1, SRT in 3, three attempts at laser ablation in 1) in five (3.3%). Eight patients (5.3%) underwent multiple treatments. No significant difference in proportions of previous treatments was seen among HH subtypes.

### 3.2 Surgical procedure and outcomes

A total of 206 SRTs were performed. Forty‐three patients (28.7%) underwent re‐SRT, with two SRTs in 33 patients (22.0%), three SRTs in seven (4.7%), and four SRTs in three (2.0%). Three patients underwent re‐SRT without GS recurrence. Numbers of burr holes, trajectories, and coagulations were highest in the MB type (Table 1).

**Figure 1:**
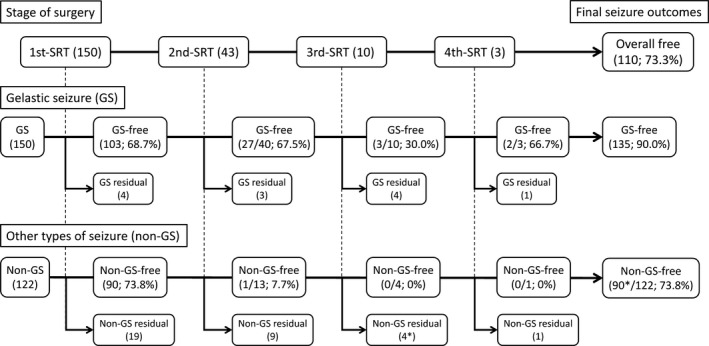
Surgical outcomes according to stage of surgery. The upper tree shows the numbers of patients in each stage of stereotactic radiofrequency thermocoagulation (SRT). Seizure outcomes are demonstrated for gelastic seizures (GSs) (middle tree) and other types of seizure (non‐GS) (lower tree) separately. Final outcomes of overall seizures, GS, and non‐GS are shown at the right edge for each tree. GS/non‐GS residual indicates patients who still experienced residual GS/non‐GS after SRT and did not undergo further operation. *One patient showed recurrence of annual generalized tonic‐clonic seizures after the third SRT

An overall seizure‐free state was finally achieved in 110 patients (73.3%). Freedom from GS was achieved by first SRT in 103 patients (68.7%), by second SRT in 30 of 40 patients (67.5%), by third SRT in three of 10 patients (30.0%), and by fourth SRT in two of three patients (66.7%). A final total of 135 patients (90.0%) became GS‐free. Although final freedom from non‐GS was achieved in 90 of 122 patients (73.8%), this was obtained from first SRT in all except one case, and re‐SRT thus appeared ineffective for recurrent non‐GS (Figure 1). In the 41 patients with residual seizures, 25 patients had residual non‐GS but freedom from GS, nine patients had residual GS but freedom from non‐GS (three of whom had GS alone), and seven patients had both residual GS and non‐GS.

Rates of final overall freedom from seizures, freedom from GS, and freedom from non‐GS did not differ among HH subtypes. Although patients with I type tended to show a relatively higher frequency of reoperation (14/35, 40%) than those with other subtypes (MU, 6/39, 15.4%; MB, 21/68, 30.9%; P, 2/8, 25.0%), no significant differences were evident (Table 1).

### 3.3 Comparison of complications between first and re‐SRT

Transient complications occurred more often with first SRT (118/150, 78.7%) than with re‐SRT (35/56, 62.5%; *P* = .02). In particular, hyponatremia was significantly more frequently observed with first SRT (52, 34.7%) compared with re‐SRT (8, 14.3%; *P* = .004). No other transient complications differed significantly between first SRT and re‐SRT. Prolonged complications likewise did not differ (first SRT, 3, 2.0%; re‐SRT, 1, 1.8%), whereas persistent complications were significantly more frequent in re‐SRT (7, 12.5%) than in first SRT (3, 2.0%; *P* = .002). Prolonged complications were as follows: with first SRT, disturbance of consciousness in two (both patients had undergone previous surgery in other institutes) and asymptomatic epidural hematoma in one patient; with re‐SRT, disturbance of consciousness in one patient. Persistent complications were as follows: with first SRT, memory disturbance in three and hypopituitarism in one patient (one patient had two complications); with re‐SRT, memory disturbance in three, hypopituitarism in three, slight hemiparesis in one, and diabetes insipidus in one patient (one patient had two complications). Weight gain, defined as a ≥5 kg increase in body weight after surgery, was significantly more frequent with first SRT (51/149, 34.2%) than with re‐SRT (10/55, 18.2%; *P* = .03). Excessive weight gain, defined as a ≥10 kg increase in body weight or a 5 kg/m^2^ increase in body mass index after surgery, was found in 22/149 (14.8%) of patients at first SRTs and in 5/55 (9.1%) of patients at re‐SRTs, showing no significant difference (Table 2).

**Table 2:** Comparison between first SRT and re‐SRT

	First SRT (n = 150)	Re‐SRT (n = 56)	OR (95% CI)	*P* value
Burr holes	2 (1‐3, 1‐2)	1 (1‐3, 1‐2)		<.001
Trajectories	4 (1‐11, 3‐6)	3 (1‐7, 2‐4)		<.001
Coagulations	8 (1‐36, 5‐14)	4 (2‐32, 3‐7.75)		<.001
Transient complications	118 (78.7%)	35 (62.5%)	0.45 (0.23‐0.88)	.02
Hyponatremia	52 (34.7%)	8 (14.3%)	0.31 (0.14‐0.71)	.004
Hyperphagia	45 (30.0%)	15 (26.8%)	0.85 (0.43‐1.70)	.65
Hyperthermia	41 (27.3%)	11 (19.6%)	0.65 (0.31‐1.38)	.26
Short memory disturbance	15 (10.0%)	5 (8.9%)	0.88 (0.31‐2.55)	.82
Hemorrhagic event^a^	9 (6.0%)	0 (0%)	0	.06
Consciousness disturbance	4 (2.7%)	2 (3.6%)	1.35 (0.24‐7.59)	.73
Prolonged complications	3 (2.0%)	1 (1.8%)	0.89 (0.09‐8.75)	.92
Persistent complications	3 (2.0%)	7 (12.5%)	7.00 (1.74‐28.12)	.002
Horner's syndrome^b^	103/121 (85.1%)	38/44 (86.4%)	1.11 (0.41‐3.00)	.84
Hormonal supplementation	2 (1.3%)	4 (7.1%)	5.69 (1.01‐32.00)	.03
Weight gain^c^	51/149 (34.2%)	10/55 (18.2%)	0.43 (0.20‐0.92)	.03
Excessive weight gain^c^	22/149 (14.8%)	5/55 (9.1%)	0.58 (0.21‐1.61)	.29

Note

Binary variables are presented as n (column %). Continuous variables are expressed as median (range, IQR).

Abbreviations: CI, confidence interval; OR, odds ratio; Re‐SRT, repeat stereotactic radiofrequency thermocoagulation; SRT, stereotactic radiofrequency thermocoagulation.

a All events were asymptomatic.

b Horner's syndrome was confirmed in 121 first SRTs and in 44 re‐SRTs.

c Body weight data were missed for one patient (both first and re‐SRT).

### 3.4 Factors correlated with recurrence and residual seizures

Univariate analyses revealed that the size and subtypes of HH, as well as surgical factors including numbers of burr holes, trajectories, and coagulations, were unrelated to any seizure outcomes. Detailed results of univariate analyses are provided in Tables S1 and S2. In univariate analyses for GS outcomes (Table 3 and Table S1), younger ages at surgery (≤8 years; *P* = .002), GS onset (≤1 year; *P* = .003) and non‐GS onset (≤4 years; *P* = .01), and shorter duration of GS (≤6 years; *P* = .002) and non‐GS (≤4 years; *P* = .01) were significantly related to GS recurrence after first SRT, while only younger age at GS onset (≤1 year; *P* = .02) correlated with final GS outcomes. Daily GS correlated with recurrence after first SRT (*P* = .02), but not with final GS outcomes. HH related to genetic syndromes correlated significantly with both recurrence (*P* = .02) and final freedom from seizures (*P* = .003). Previous treatments affected neither recurrence of GS nor persistence of GS in the final follow‐up.

**Table 3:** Uni‐ and multivariate analyses of clinical factors for GS outcomes

GS outcomes after first SRT						
	GS‐free	GS recurrence	Univariate analysis		Multivariate analysis	
			OR (95% CI)	*P* value	OR (95% CI)	*P* value
Number	103	47				
Age at first SRT						
≤8 y	44 (57.1%)	33 (42.9%)	0.32 (0.15‐0.66)	.002	1.01 (0.14‐7.26)	.99
>8 y	59 (80.8%)	14 (19.2%)				
Age at GS onset						
≤1 y	60 (60.6%)	39 (39.4%)	0.29 (0.12‐0.67)	.003	4.91 (1.49‐16.24)	.009
>1 y	43 (84.3%)	8 (15.7%)				
Duration of GS						
≤6 y	44 (57.1%)	33 (42.9%)	0.32 (0.15‐0.66)	.002	2.43 (0.36‐16.60)	.36
>6 y	59 (80.8%)	14 (19.2%)				
Age at non‐GS onset						
≤4 y	39 (59.1%)	27 (40.9%)	0.35 (0.16‐0.80)	.01	1.57 (0.43‐5.67)	.49
>4 y	45 (80.4%)	11 (19.6%)				
Duration of non‐GS						
≤4 y	35 (58.3%)	25 (41.7%)	0.37 (0.15‐0.66)	.01	1.27 (0.35‐4.67)	.72
>4 y	49 (79.0%)	13 (21.0%)				
Behavioral disorder						
(+)	55 (67.9%)	26 (32.1%)	1.08 (0.54‐2.16)	.83	0.53 (0.17‐1.59)	.25
(−)	48 (67.9%)	21 (30.4%)				
Intellectual disability						
(+)	55 (75.3%)	26 (24.7%)	0.54 (0.27‐1.15)	.09	0.49 (0.18‐1.37)	.18
(−)	48 (62.3%)	29 (37.6%)				
Genetic syndrome						
(+)	4 (36.4%)	7 (63.6%)	4.33 (1.20‐15.61)	.02	4.61 (0.63‐33.95)	.13
(−)	99 (71.2%)	40 (28.8%)				
Previous open surgery						
(+)	18 (81.8%)	4 (18.2%)	0.44 (0.14‐1.38)	.15	0.44 (0.10‐1.98)	.29
(−)	85 (66.4%)	43 (33.6%)				
Previous GKS						
(+)	13 (59.1%)	9 (40.9%)	1.64 (0.65‐4.16)	.30	0.82 (0.21‐3.22)	.78
(−)	90 (70.3%)	38 (29.7%)				
Multiple treatments						
(+)	5 (62.5%)	3 (37.5%)	1.34 (0.31‐5.84)	.70	3.42 (0.44‐26.15)	.24
(−)	98 (69.0%)	44 (31.0%)				

Final GS outcomes						
	GS‐free	GS residual	Univariate analysis		Multivariate analysis	
			OR (95% CI)	*P* value	OR (95% CI)	*P* value
Number	135	15				
Age at first SRT						
≤8 y	66 (85.7%)	11 (14.3%)	0.35 (0.11‐1.15)	.07	0.80 (0.03‐25.43)	.90
>8 y	69 (94.5%)	4 (5.5%)				
Age at GS onset						
≤1 y	85 (85.9%)	14 (14.1%)	0.12 (0.02‐0.95)	.02	8.29 (0.75‐91.43)	.08
>1 y	50 (98.0%)	1 (2.0%)				
Duration of GS						
≤6 y	66 (85.7%)	11 (14.3%)	0.35 (0.11‐1.15)	.07	8.02 (0.27‐234.71)	.23
>6 y	69 (94.5%)	4 (5.5%)				
Age at non‐GS onset						
≤4 y	56 (84.9%)	10 (15.1%)	0.32 (0.08‐1.22)	.08	0.73 (0.10‐5.37)	.75
>4 y	53 (94.6%)	3 (5.4%)				
Duration of non‐GS						
≤4 y	53 (88.3%)	7 (11.7%)	0.81 (0.26‐2.57)	.72	0.12 (0.01‐1.17)	.07
>4 y	56 (90.3%)	6 (9.7%)				
Behavioral disorder						
(+)	70 (86.4%)	11 (13.6%)	2.55 (0.78‐7.03)	.11	2.67 (0.51‐14.01)	.25
(−)	65 (94.2%)	4 (5.8%)				
Intellectual disability						
(+)	55 (75.3%)	18 (24.7%)	0.49 (0.16‐1.52)	.21	0.27 (0.06‐1.25)	.09
(−)	67 (87.0%)	10 (13.0%)				
Genetic syndrome						
(+)	7 (63.6%)	4 (36.4%)	6.65 (1.68‐26.28)	.003	5.47 (0.79‐38.08)	.09
(−)	128 (92.1%)	11 (7.9%)				
Previous open surgery						
(+)	21 (95.4%)	1 (4.6%)	0.39 (0.05‐3.11)	.36	0.35 (0.03‐3.74)	.38
(−)	114 (89.1%)	14 (10.9%)				
Previous GKS						
(+)	20 (90.9%)	2 (9.1%)	0.89 (0.19‐4.22)	.88	0.80 (0.11‐6.06)	.83
(−)	115 (89.8%)	13 (10.2%)				
Multiple treatments						
(+)	8 (100.0%)	0	0	.33	<0.001	.99
(−)	127 (89.4%)	15 (10.6%)				

Abbreviations: CI, confidence interval; GKS, gamma knife radiosurgery; GS, gelastic seizure; Non‐GS, other types of seizure; OR, odds ratio; SRT, stereotactic radiofrequency thermocoagulation.

In contrast (Table 4 and Table S2), longer duration of GS (>6 years; *P* = .02) and non‐GS (>4 years; *P* = .02), daily non‐GS (*P* < .001), tonic seizures (*P* < .001), atonic seizures (*P* = .01), ID (*P* < .001), genetic syndromes (*P* = .005 and *P* < .001, respectively), GKS (*P* = .01), and multiple previous treatments (*P* < .001) correlated significantly both with non‐GS recurrences after first SRT and final residual non‐GS.

**Table 4:** Uni‐ and multivariate analyses of clinical factors for non‐GS outcomes

Non‐GS outcomes after first SRT						
	Non‐GS‐free	Non‐GS recurrence	Univariate analysis		Multivariate analysis	
			OR (95% CI)	*P* value	OR (95% CI)	*P* value
Number	90	32				
Age at first SRT						
≤8 y	45 (81.8%)	10 (18.2%)	2.2 (0.94‐5.17)	.07	1.06 (0.13‐8.22)	.96
>8 y	45 (67.2%)	22 (32.8%)				
Age at GS onset						
≤1 y	55 (68.8%)	25 (31.2%)	2.27 (0.89‐5.81)	.08	1.83 (0.44‐7.64)	.41
>1 y	35 (83.3%)	7 (16.7%)				
Duration of GS						
≤6 y	46 (83.6%)	9 (16.4%)	2.67 (1.11‐6.41)	.02	0.49 (0.06‐4.17)	.51
>6 y	44 (65.7%)	23 (34.3%)				
Age at non‐GS onset						
≤4 y	49 (74.2%)	17 (25.8%)	1.05 (0.47‐2.37)	.90	0.20 (0.04‐1.02)	.05
>4 y	41 (73.2%)	15 (26.8%)				
Duration of non‐GS						
≤4 y	50 (83.3%)	10 (16.7%)	2.75 (1.17‐6.47)	.02	0.93 (0.17‐5.03)	.93
>4 y	40 (64.5%)	22 (35.5%)				
CPS						
(+)	58 (76.3%)	18 (23.7%)	0.71 (0.31‐1.61)	.41	0.64 (0.16‐2.63)	.54
(−)	32 (69.6%)	14 (30.4%)				
TS						
(+)	27 (54.0%)	23 (46.0%)	5.96 (2.44‐14.56)	<.001	4.89 (1.45‐16.44)	.01
(−)	63 (87.5%)	9 (12.5%)				
GTCS						
(+)	43 (70.5%)	18 (29.5%)	1.41 (0.62‐3.16)	.41	0.39 (0.11‐1.38)	.15
(−)	47 (77.1%)	14 (22.9%)				
Behavioral disorder						
(+)	48 (72.7%)	18 (27.3%)	1.13 (0.50‐2.53)	.78	0.40 (0.09‐1.73)	.22
(−)	42 (75.0%)	14 (25.0%)				
Intellectual disability						
(+)	35 (55.6%)	28 (44.4%)	11.00 (3.55‐34.06)	<.001	17.35 (3.44‐87.47)	<.001
(−)	55 (93.2%)	4 (6.8%)				
Genetic syndrome						
(+)	2 (28.6%)	5 (71.4%)	8.15 (1.50‐44.41)	.005	23.67 (2.16‐259.09)	.001
(−)	88 (76.5%)	27 (23.5%)				
Previous open surgery						
(+)	14 (66.7%)	7 (33.3%)	1.52 (0.55‐4.19)	.42	0.78 (0.15‐4.06)	.76
(−)	76 (75.3%)	25 (24.7%)				
Previous GKS						
(+)	9 (50.0%)	9 (50.0%)	3.52 (1.25‐9.90)	.01	0.67 (0.09‐4.87)	.69
(−)	81 (77.9%)	23 (22.1%)				
Multiple treatments						
(+)	1 (12.5%)	7 (87.5%)	24.92 (2.93‐212.18)	<.001	22.74 (1.01‐511.80)	.05
(−)	89 (78.1%)	25 (21.9%)				

Final GS outcomes						
	GS‐free	GS residual	Univariate analysis		Multivariate analysis	
			OR (95% CI)	*P* value	OR (95% CI)	*P* value
Number	135	15				
Age at first SRT						
≤8 y	45 (81.8%)	10 (18.2%)	2.2 (0.94‐5.17)	.07	0.93 (0.12‐7.15)	.94
>8 y	45 (67.2%)	22 (32.8%)				
Age at GS onset						
≤1 y	55 (68.8%)	25 (31.2%)	2.27 (0.89‐5.81)	.08	1.73 (0.43‐6.93)	.44
>1 y	35 (83.3%)	7 (16.7%)				
Duration of GS						
≤6 y	46 (83.6%)	9 (16.4%)	2.67 (1.11‐6.41)	.02	0.53 (0.06‐4.41)	.56
>6 y	44 (65.7%)	23 (34.3%)				
Age at non‐GS onset						
≤4 y	49 (74.2%)	17 (25.8%)	1.05 (0.47‐2.37)	.90	0.21 (0.04‐1.05)	.06
>4 y	41 (73.2%)	15 (26.8%)				
Duration of non‐GS						
≤4 y	50 (83.3%)	10 (16.7%)	2.75 (1.17‐6.47)	.02	0.76 (0.14‐4.22)	.75
>4 y	40 (64.5%)	22 (35.5%)				
CPS						
(+)	58 (76.3%)	18 (23.7%)	0.71 (0.31‐1.61)	.41	0.86 (0.22‐3.45)	.84
(−)	32 (69.6%)	14 (30.4%)				
TS						
(+)	27 (54.0%)	23 (46.0%)	5.96 (2.44‐14.56)	<.001	4.62 (1.35‐15.82)	.01
(−)	63 (87.5%)	9 (12.5%)				
GTCS						
(+)	42 (68.9%)	19 (31.1%)	1.67 (0.74‐3.79)	.21	0.63 (0.18‐2.22)	
(−)	48 (78.7%)	13 (21.3%)				
Behavioral disorder						
(+)	47 (71.2%)	19 (28.9%)	1.34 (0.59‐3.03)	.49	0.74 (0.18‐3.08)	.68
(−)	43 (76.8%)	13 (23.2%)				
Intellectual disability						
(+)	36 (57.1%)	27 (42.9%)	8.10 (2.85‐23.00)	<.001	8.48 (1.88‐38.30)	.006
(−)	54 (91.5%)	5 (8.5%)				
Genetic syndrome						
(+)	1 (14.3%)	6 (85.7%)	20.54 (2.36‐178.38)	<.001	68.52 (4.61‐1018.03)	.002
(−)	89 (77.4%)	26 (22.6%)				
Previous open surgery						
(+)	14 (66.7%)	7 (33.3%)	1.52 (0.55‐4.19)	.42	0.57 (0.11‐3.02)	
(−)	76 (75.3%)	25 (24.7%)				
Previous GKS						
(+)	9 (50.0%)	9 (50.0%)	3.52 (1.25‐9.90)	.01	0.52 (0.06‐4.28)	.55
(−)	81 (77.9%)	23 (22.1%)				
Multiple treatments						
(+)	1 (12.5%)	7 (87.5%)	24.92 (2.93‐212.18)	<.001	30.14 (1.29‐705.86)	.03
(‐)	89 (78.1%)	25 (21.9%)				

Abbreviations: CI, confidence interval; CPS, complex partial seizure; GKS, gamma knife surgery; GS, gelastic seizure; GTCS, generalized tonic‐clonic seizure; Non‐GS, other types of seizure; OR, odds ratio; SRT, stereotactic radiofrequency thermocoagulation; TS, tonic seizure.

Multivariate analyses for GS outcomes (Table 3) revealed that only age at GS onset (≤1 year; *P* = .009) correlated with GS recurrence after first SRT, whereas no variables were found to affect final GS outcomes. On the other hand, tonic seizure (*P* = .01), ID (*P* < .001 and = .006, respectively), and genetic syndromes (*P* = .001 and .002, respectively) correlated with both non‐GS recurrence after first SRT and final outcome. In addition, multiple treatments (*P* = .03) correlated with final non‐GS outcomes (Table 4).

## 4 DISCUSSION

The present study demonstrated that SRT still achieved favorable seizure outcomes, as previously reported,^9^ even after the number of patients reached 150. Final freedom from overall seizures, GS, and non‐GS was achieved in 73%, 90%, and 74% of patients, respectively. About 30% of patients underwent re‐SRT for recurrent GS, and re‐SRT had been effective in stages. Transient and prolonged complications were not increased with re‐SRT compared with first SRT, while persistent complications were significantly more frequent with re‐SRT. Among the factors associated with seizure outcomes, there was no factor correlated with final GS outcomes, whereas age factors as well as patient conditions, such as seizure semiology and previous treatments, correlated with non‐GS outcomes. Genetic syndromes were related to both GS and non‐GS. Neither HH morphology nor surgical procedures correlated with seizure outcomes.

### 4.1 Significance and feasibility of reoperation

**Figure 2:**
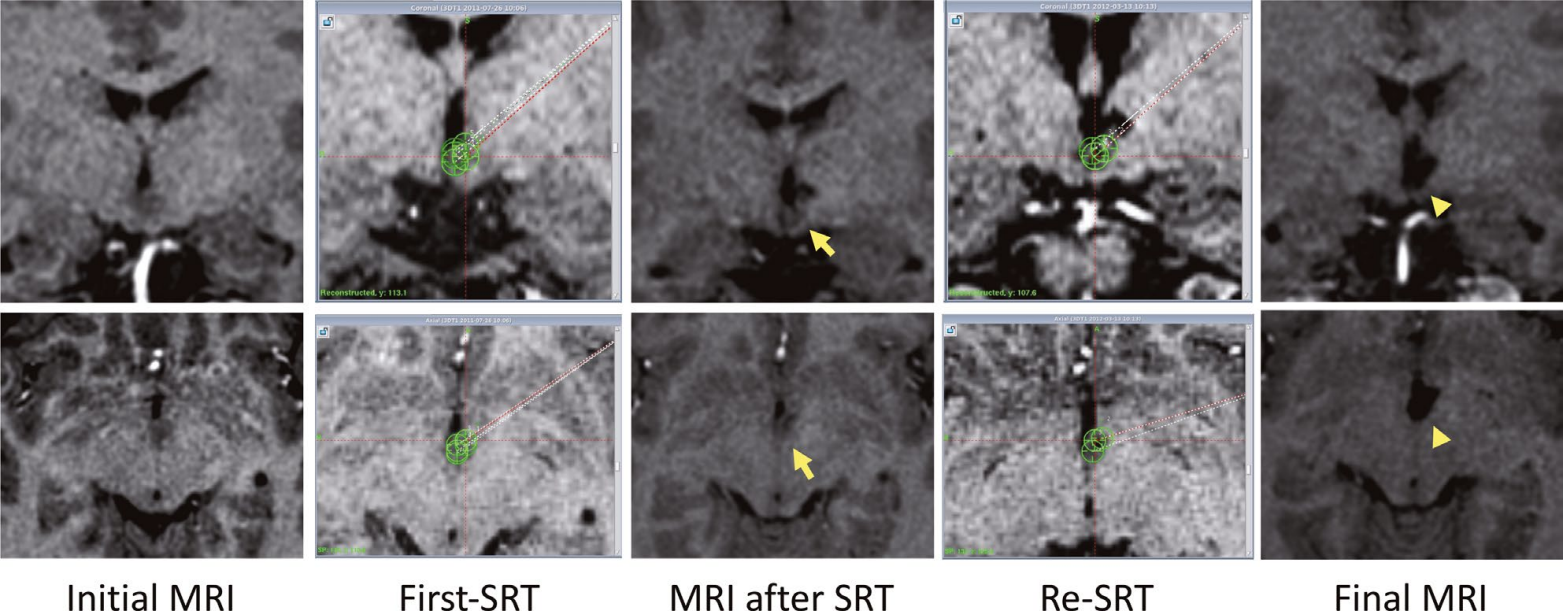
An illustrative case. A 13‐year‐old girl who experienced very frequent GS and medically controlled complex partial, tonic, and generalized tonic‐clonic seizures underwent SRT (4 trajectories/5 coagulations) for intrahypothalamic‐type HH (maximum diameter, 9 mm). GS recurred 3 months after surgery, and follow‐up MRI revealed incomplete coagulation and a residual part at the bottom of the HH attached to the left hypothalamus (arrows). Re‐SRT (2 trajectories/3 coagulations) performed 8 months after the first SRT eliminated recurrent GS for more than 5 y without any persistent complications. Follow‐up MRI showed complete disconnection (arrowheads). Upper row, coronal images; lower row, axial images

Our results support the notion that re‐SRT is effective for recurrent GS after SRT, as well as that after failed previous treatments. Our rationale for SRT is complete disconnection between HH and the hypothalamus.^9^ Even tiny residual hamartoma could cause recurrence of GS. Moreover, bilateral attachment probably generates GS by propagation through bilateral attachment.^19^ Incomplete disconnection in previous surgeries has the potential to cause recurrent GS. Re‐SRT targeting the residual part could eliminate GS. That is why our strategy for SRT is complete disconnection between HH and the hypothalamus to the fullest extent possible (Figure 2, Figure S1).

The reoperation rate of 28.7% in the present study was relatively high. Although large cohorts for studies on laser ablation remain difficult to obtain, recent reports have shown reoperation rates for multiple ablations of 23%^20^ and 17%.^21^ These series achieved favorable GS outcomes (93% and 80% achieved final freedom from GS) comparable to the results of the present study. Both stereotactic procedures are minimally invasive, providing ease of reoperation and further freedom from seizures.

A small number of reports have mentioned the significance of reoperation. Reoperation has been recommended for some selected patients.^15^, ^16^ However, the reoperation was usually combined with multiple types of procedure. A reoperation means the patient must undergo an additional invasive surgery. Furthermore, the combination of multiple procedures may also cause multiple additional complications specific to the procedures. The pterional approach is known to show a relatively high prevalence of risks of stroke or cranial nerve injury,^7^ and the interhemispheric approach carries a risk of memory impairment.^13^, ^22^ Endoscopic surgery is also accompanied by a risk of memory impairment.^13^ There is little risk in gamma knife radiosurgery,^23^, ^24^, ^25^ but the possibility of late adverse effects should not be disregarded.^26^, ^27^

Stereotactic radiofrequency thermocoagulation is a minimally invasive technique and has a relatively low risk of complications. The present study demonstrated that re‐SRT showed a lower incidence of transient and prolonged complications compared with first SRT, except for the relatively higher incidence of persistent complications. However, these incidences (re‐SRT, 7/56, 12.5%; overall, 10/206, 4.9%) were not so high compared with other treatments, such as the pterional approach,^7^, ^28^ interhemispheric approach,^13^, ^22^ or endoscopic approach.^13^, ^29^ Because of these tolerable complication profiles as well as the effectiveness, re‐SRT should be considered for patients with recurrent GS.

### 4.2 Clinical factors for GS outcomes

Age factors were significantly related to GS outcomes. Younger age at surgery, younger onset of seizures, and shorter duration of seizures correlated with GS recurrence after first SRT, but no factor was related to final GS outcomes. Younger age at GS onset may reflect the stronger epileptogenesis of HH itself, regardless of size, shape, or location. HH with stronger epileptogenesis may be likely to result in unfavorable outcomes following incomplete surgery. As our surgery can be applied to every HH consistently under the rationale of complete disconnection, the morphology of HH and surgical procedures are not considered to be important factors for seizure outcomes. On the other hand, GS can be cured by SRT at any age. The maximum age at surgery in this series was 50 years, for a male patient who achieved freedom from GS. Multivariate analyses indicated that only age at GS onset correlated with GS recurrence, with no factors related to final GS outcomes. The epileptogenic mechanisms involved in GS might depend robustly on the connection between HH and the hypothalamus for ages. Complete disconnection of HH therefore provides freedom from GS at any age.

### 4.3 Clinical factors for non‐GS outcomes

In contrast, longer duration of seizures was correlated significantly with both recurrence after first SRT and final outcomes of non‐GS in univariate analyses. However, multivariate analyses revealed previous multiple treatments were a significant factor instead of longer duration of seizures. Non‐GS is thought to be attributed to secondary epileptogenesis, which involves a different epileptogenic mechanism from the original epileptogenic network generating GS.^1^, ^30^, ^31^ Actually in the present study, most patients (106/122, 95.1%) presented with initial GS followed by non‐GS. Longer duration of epilepsy might result in the independence of secondary epileptogenesis,^31^, ^32^ or multiple previous treatments might complicate the epileptogenic network by time‐consuming. Once the secondary epileptogenesis becomes independent from the original epileptogenic network, surgical intervention for the original network would not achieve seizure control.^31^ As a previous report^9^ and this study demonstrated, re‐SRT was not effective for recurrent non‐GS. If non‐GS remains after re‐SRT, other treatment options would be considered, including powerful pharmacotherapies, vagus nerve stimulation, or others. However, no supporting data have been accumulated regarding the effectiveness of these additional treatments. The effects of age on seizure recurrence after surgical treatment have not been documented in previous studies.^9^

Another important factor affecting non‐GS outcomes was genetic syndromes. Genetic factors were significantly related to non‐GS outcomes in both uni‐ and multivariate analyses. In a previous report,^33^ contradictory results of better seizure outcomes were demonstrated in patients with Pallister‐Hall syndrome than in those with isolated HH. Although it is difficult to compare our study and a previous report involving a different population, genetic factors have been assumed to have impacts on the developing brain. ID was also related to non‐GS outcomes in both uni‐ and multivariate analyses. Berkovic et al^8^ once used “treatable epileptic encephalopathy” for HH syndrome with favorable seizure outcomes as well as functional outcomes. However, our series contained patients with unfavorable seizure outcomes and severe ID even after SRT. The severe form of HH syndrome with treatment‐resistant non‐GS and severe ID may be considered to relate to a developmental and epileptic encephalopathy,^34^ which may be termed “HH encephalopathy.”

### 4.4 Indications and optimal timing of SRT

Compared with other treatment options, SRT could be a single option for HH treatment without limitations on the size, shape, or location of the HH,^9^, ^18^ contradicting a past article suggesting that no single neurosurgical approach is effective for all forms of HH.^14^ Moreover, the present study demonstrated the effectiveness of re‐SRT for patients with recurrent GS, as well as for those with failed previous treatments. On the other hand, the present study also demonstrated that most cases of recurrent non‐GS did not react to re‐SRT. If patients experience recurrence of non‐GS alone instead of GS, they are unlikely to be candidates for re‐SRT.

Age was indicated as an important factor for recurrence or final outcomes of seizures. Younger age was an important factor for GS recurrences. HH with early onset of GS may require drastic treatment. In contrast, older age at surgery and longer duration of epilepsy were likely to be related to non‐GS outcomes. Previous treatments, especially multiple treatments, were also significant negative factors. Because repeated multiple ineffective treatments result in wasted time, late indication of SRT appears unfavorable, especially for patients with non‐GS. Early surgical indications for patients with non‐GS would thus increasingly improve surgical outcomes.

Our proposals for the indications and optimal timing of SRT are as follows: Patients with only GS may not require any haste in undergoing SRT unless the GS are too frequent and disturb daily life. Patients with non‐GS should be considered for early SRT where possible. Patients accompanied by ID are also candidates for early surgery, because recovery from ID is likely to be obtained in those with shorter duration of epilepsy.^10^ Time‐wasting palliative treatments or time‐consuming by repeated ineffective multiple treatments should be avoided.

## 5 CONCLUSIONS

The present study demonstrated that re‐SRT can be performed with potential benefits in terms of freedom from seizures, albeit with increased risk of persistent complications, and finally provided favorable seizure outcomes in this large series of 150 patients. Repeated ineffective treatments should be avoided, and an early indication of SRT is recommended.

## CONFLICT OF INTEREST

None of the authors has any conflict of interest to disclose. We confirm that we have read the Journal's position on issues involved in ethical publication and affirm that this report is consistent with those guidelines.
