# Supplementary material for: Repeat stereotactic radiofrequency thermocoagulation in patients with hypothalamic hamartoma and seizure recurrence
Source: Epilepsia Open. 2020 Jan 18;5(1):107–20. doi: 10.1002/epi4.12378 (PMC7049799; doi:10.1002/epi4.12378)
Supplement: Supplementary file 1 [file EPI4-5-107-s001.pdf]

Figure S1. Illustrative cases

Case: A 3-year-old girl

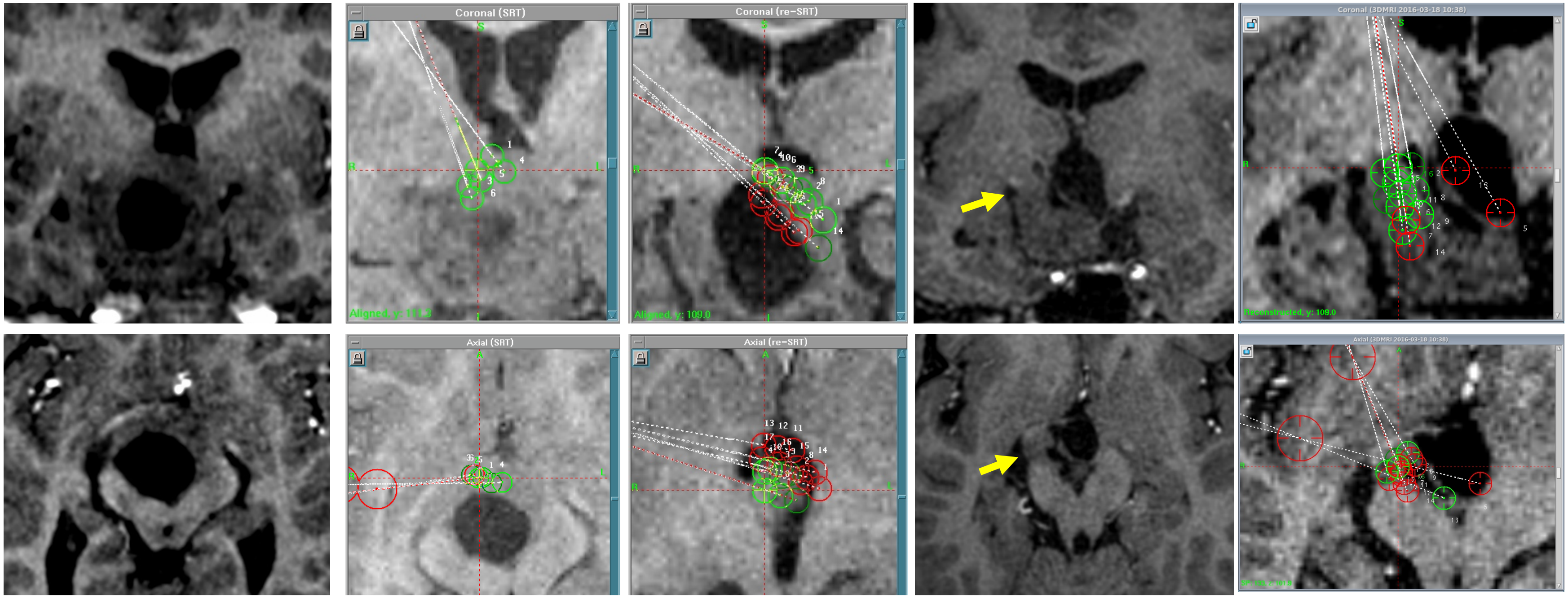

Initial MRI | First SRT | Second SRT | MRI after second SRT | Third SRT

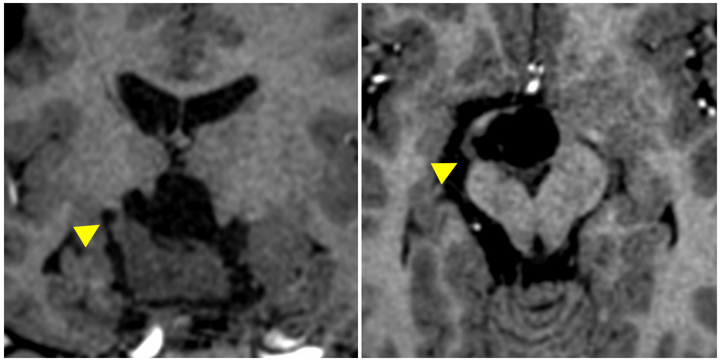

Final MRI

Case: A 3-year-old girl (cont.)

A 3-years-old girl with giant hypothalamic hamartoma (HH) (maximum diameter, 50mm) containing a large cyst experienced very frequent gelastic seizures (GS) and a few instances of generalized tonic-clonic seizures. She had undergone stereotactic radiofrequency thermocoagulation (SRT) twice by the age of 3 years old. The first and second SRT consisted of 3 trajectories/6 coagulations and 5 trajectories/17 coagulations, respectively. GS recurred 5 years after second SRT and MRI revealed residual HH in the lateral part (arrows). The third SRT was conducted to target the residual part with 6 trajectories/14 coagulations. She was free from GS for 2 years after final SRT without any persistent complications. Final MRI showed complete disconnection (arrowheads).

- Upper row, coronal images; middle row, axial images; lower left, coronal image; lower right, axial image

Case: A 5-year-old boy

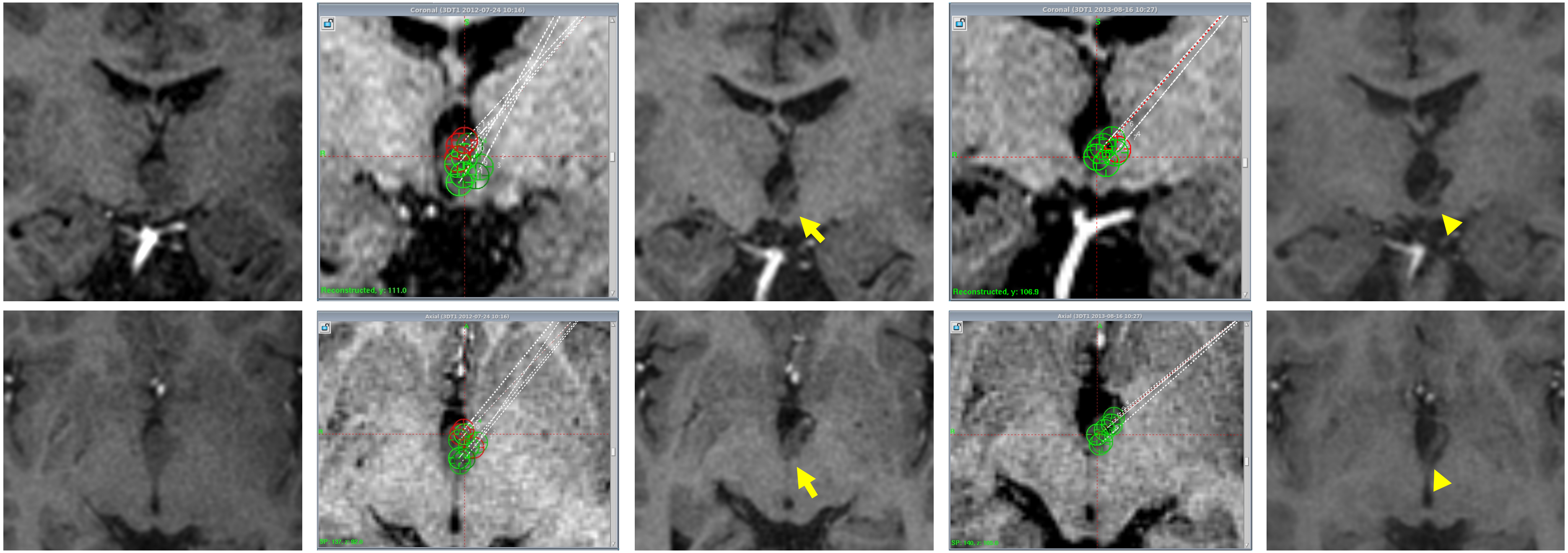

Initial MRI | First SRT | MRI after SRT | Re-SRT | Final MRI

A 5-year-old boy who experienced daily GS and weekly generalized tonic-clonic seizures (GTCS) underwent SRT (5 trajectories/10 coagulations) for intrahypothalamic-type HH (maximum diameter, 13 mm). GS recurred 2 weeks after surgery and follow-up MRI revealed a residual part in the posterior region of the HH (arrows). Re-SRT (3 trajectories/6 coagulations) was performed 1 year after the first SRT and GS was eliminated without any persistent complications. Follow-up MRI showed complete disconnection (arrowheads).

- Upper row, coronal images; middle row, axial images (following slides use the same format)

Case: A 3-year-old boy

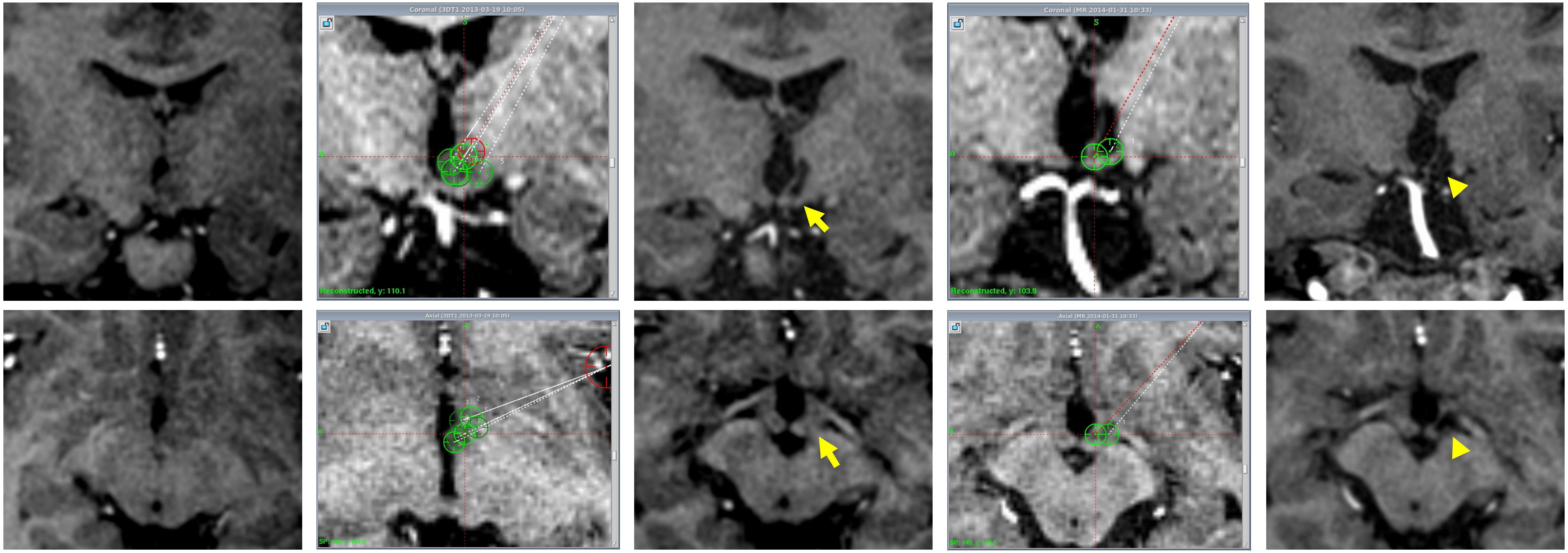

Initial MRI | First-SRT | MRI after SRT | Re-SRT | Final MRI

A 3-year-old boy who experienced very frequent GS underwent SRT (4 trajectories/6 coagulations) for intrahypothalamic-type HH (maximum diameter, 12 mm). GS recurred 3 months after surgery. Although follow-up MRI showed almost complete disconnection, a very tiny residual attachment was suspected in the postero-lateral part of the HH (arrows). As monthly frequency became weekly, re-SRT (2 trajectories/2 coagulations) targeting the tiny residual part was conducted 8 months after the first SRT. MRI after re-SRT revealed complete disconnection (arrow heads), and GS was completely eliminated for 5 years without any persistent complications.

Case: A 5-year-old girl

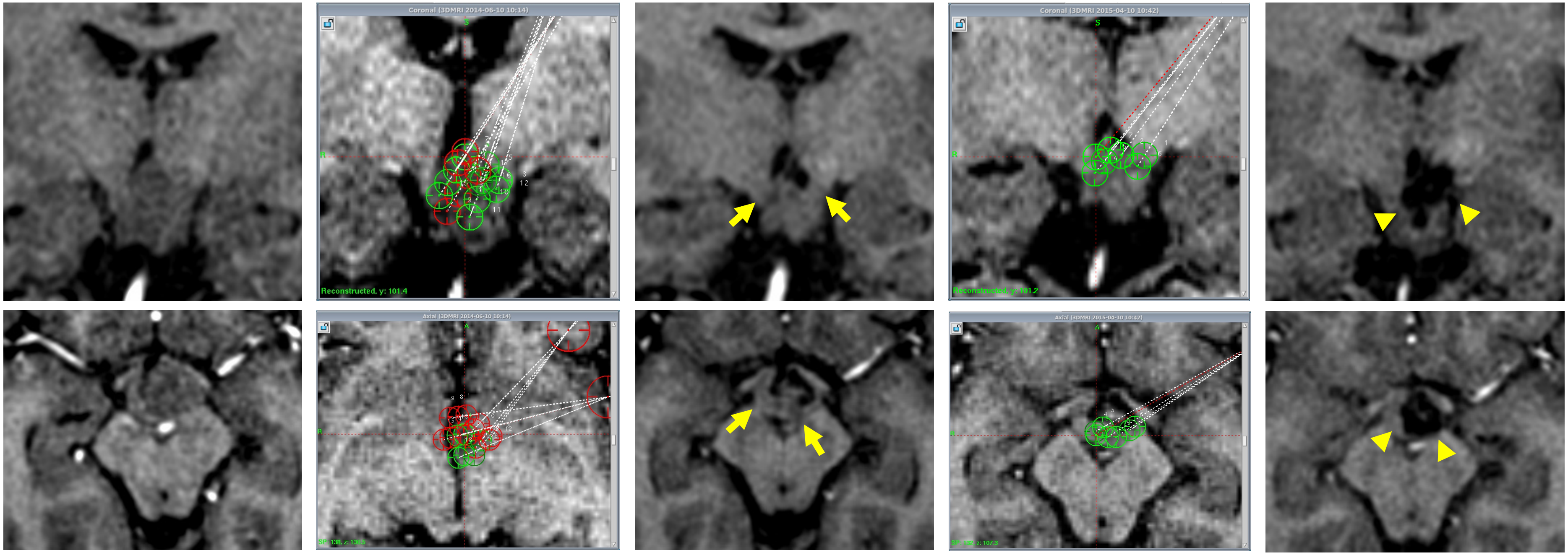

Initial MRI | First-SRT | MRI after SRT | Re-SRT | Final MRI

A 5-year-old girl who presented with daily GS and monthly complex partial and tonic seizures showed a mixed-type HH with bilateral attachments (maximum diameter, 21 mm). SRT (7 trajectories/17 coagulations) failed to achieve complete disconnection of the attachment (arrows), and GS recurred 1 month after surgery. Excessive body weight gain was also observed. Re-SRT (5 trajectories/7 coagulations) for residual bilateral attachments was performed 10 months after the first SRT. GS was completely eliminated, but excessive body weight gain was unchanged.

Case: A 16-year-old girl

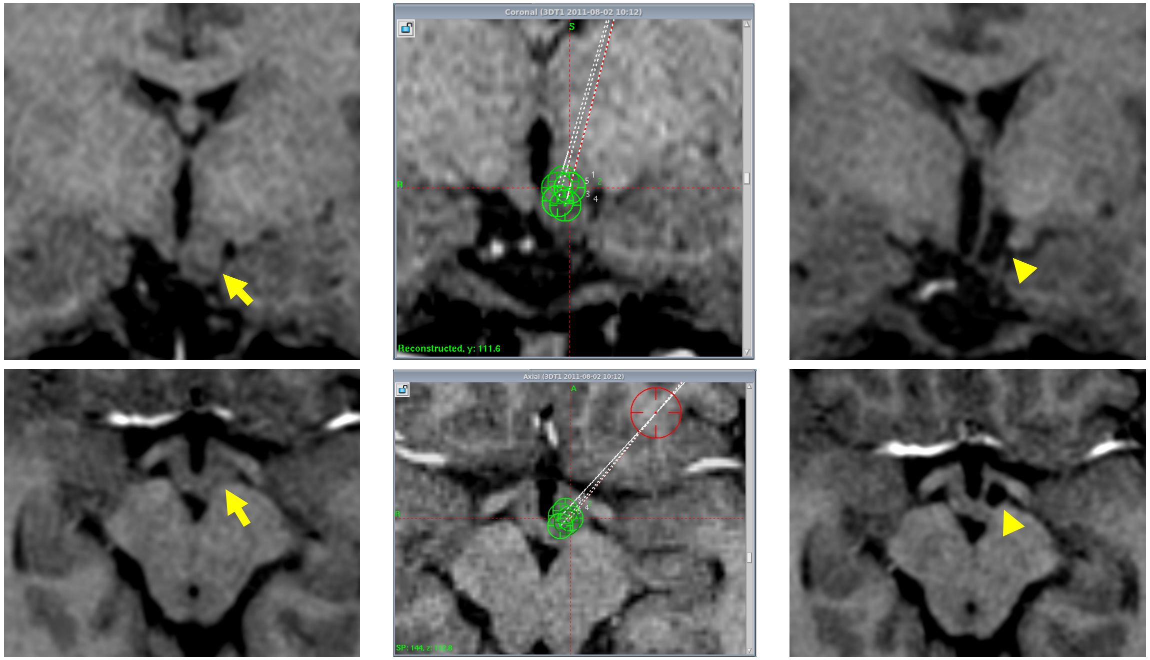

Initial MRI SRT Final MRI

A 16-year-old girl had undergone partial resection via the right pterional approach at 11 years old, resulting in no change of GS. MRI showed residual HH attaching to the left hypothalamus (arrows). SRT (3 trajectories/5 coagulations) was performed, and she achieved freedom from GS for 5 years.

Case: A 42-year-old man

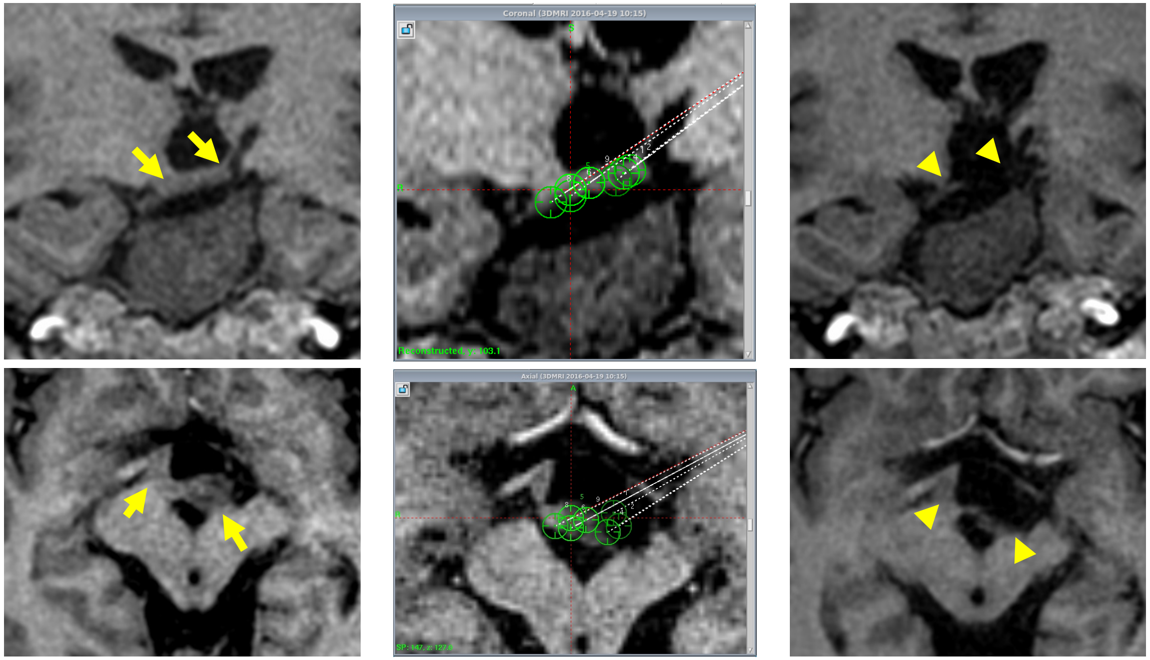

Initial MRI SRT Final MRI

A 42-year-old man had shown GS since infancy and HH had been diagnosed at 20 years old. Disconnection surgery was performed via the right subtemporal approach, reducing GS by more than 90%, but mild right hemiparesis and memory disturbance remained. Follow-up MRI showed well-disconnected HH, but also suggested a slight residual component attached bilaterally to the hypothalamus (arrows). Annual non-gelastic seizures remained for years, but the frequency gradually increased from 40 years old. Daily GS and weekly complex partial seizure proved refractory to medical treatment, then SRT (4 trajectories/7 coagulations) was performed. All seizures were eliminated after SRT except for one GTCS when he neglected to take the prescribed anti-epileptic drug.
