# Supplementary material for: Repeat stereotactic radiofrequency thermocoagulation in patients with hypothalamic hamartoma and seizure recurrence
Source: Epilepsia Open. 2020 Jan 18;5(1):107–20. doi: 10.1002/epi4.12378 (PMC7049799; doi:10.1002/epi4.12378)
Supplement: Supplementary file 2 [file EPI4-5-107-s002.docx]

Table S1. Univariate analyses of clinical factors for GS outcomes

|  | | | | GS outcomes after first SRT | | | | Final GS outcomes | | | |
| --- | --- | --- | --- | --- | --- | --- | --- | --- | --- | --- | --- |
|  | | | | GS-free | GS recurrence | OR (95%CI) | *P* value | GS-free | GS residual | OR (95%CI) | *P* value |
| Number | | | | 103 | 47 |  |  | 135 | 15 |  |  |
|  | | | | | | | | | | | |
| Sex | Male | | | 64 (69.6%) | 28 (30.4%) | 0.90  (0.44–1.82) | 0.77 | 84 (87.9%) | 8 (8.7%) | 0.69  (0.24–2.03) | 0.50 |
|  | Female | | | 39 (67.2%) | 19 (32.8%) |  |  | 51 (87.9%) | 7 (12.1%) |  |  |
| Age at first-SRT, y  (median, IQR) | | | | 1.8–50  (10, 5–17) | 1.7–50  (5, 3–13) |  |  | 1.8–50  (9, 4–16) | 1.7–42  (4, 2–20) |  |  |
|  | **≤ 8 y at first SRT** | | | **44 (57.1%)** | **33 (42.9%)** | **0.32**  (0.15–0.66) | **0.002** | 66 (85.7%) | 11 (14.3%) | 0.35  (0.11–1.15) | 0.07 |
|  | > 8 y at first SRT | | | 59 (80.8%) | 14 (19.2%) |  |  | 69 (94.5%) | 4 (5.5%) |  |  |
| Age at GS onset, y  (median, IQR) | | | | 0–10  (1, 0.1–2.5) | 0–11  (0.3, 0–1) |  |  | 0–11  (0.8, 0–2) | 0–4  (0.3, 0–1) |  |  |
|  | **≤ 1 y at GS onset** | | | **60 (60.6%)** | **39 (39.4%)** | **0.29**  **(0.12–0.67)** | **0.003** | **85 (85.9%)** | **14 (14.1%)** | **0.12**  **(0.02–0.95)** | **0.02** |
|  | > 1 y at GS onset | | | 43 (84.3%) | 8 (15.7%) |  |  | 50 (98.0%) | 1 (2.0%) |  |  |
| Duration of GS, y  (median, IQR) | | | | 0.7–45  (8, 3–15) | 0–47  (4, 2.5–12) |  |  | 0–47  (6.5, 3–14.5) | 1–42  (3, 1.7–19.3) |  |  |
|  | **≤ 6 y of GS** | | | **44 (57.1%)** | **33 (42.9%)** | **0.32**  **(0.15–0.66)** | **0.002** | 66 (85.7%) | 11 (14.3%) | 0.35  (0.11–1.15) | 0.07 |
|  | > 6 y of GS | | | 59 (80.8%) | 14 (19.2%) |  |  | 69 (94.5%) | 4 (5.5%) |  |  |
| Age at non-GS onset, y  (median, IQR) | | | | 0–28  (5, 2–9) | 0–14  (2, 1–5.25) |  |  | 0–28  (4, 1.85–9) | 0–11  (1.8, 1.2–6) |  |  |
|  | **≤ 4 y at non-GS onset** | | | **39 (59.1%)** | **27 (40.9%)** | **0.35**  **(0.16–0.80)** | **0.01** | 56 (84.9%) | 10 (15.1%) | 0.32  (0.08–1.22) | 0.08 |
|  | > 4 y at non-GS onset | | | 45 (80.4%) | 11 (19.6%) |  |  | 53 (94.6%) | 3 (5.4%) |  |  |
| Duration of non-GS, y  (median, IQR) | | | | 0–45  (5.25, 2–11.95) | 0–39  (2.5, 1–11.5) |  |  | 0–45  (4.7, 1.75–11.1) | 0–32  (1.7, 0.35–19.45) |  |  |
|  | **≤ 4 y of non-GS** | | | **35 (58.3%)** | **25 (41.7%)** | **0.37**  **(0.15–0.66)** | **0.01** | 53 (88.3%) | 7 (11.7%) | 0.81  (0.26–2.57) | 0.72 |
|  | > 4 y of non-GS | | | 49 (79.0%) | 13 (21.0%) |  |  | 56 (90.3%) | 6 (9.7%) |  |  |
|  | | | | | | | | | | | |
| Maximum diameter of HH, mm | | | | 4.5–42  (15, 10–22) | 7–80  (13, 10–23) |  |  | 4.5–80  (15, 10–23) | 7–43  (13, 10–22 |  |  |
|  | ≤ 15 mm | | | 52 (66.7%) | 26 (33.3%) | 0.82  (0.41–1.65) | 0.58 | 70 (89.7%) | 8 (10.3%) | 0.94  (0.32–2.75) | 0.91 |
|  | > 15 mm | | | 51 (70.8%) | 21 (29.2%) |  |  | 65 (90.3%) | 7 (9.7%) |  |  |
| HH subtypes | | | |  |  |  | 0.22 |  |  |  | 0.75 |
|  | Parahypothalamic | | | 6 (75.0%) | 2 (25.0%) |  |  | 8 (100%) | 0 |  |  |
|  | Intrahypothalamic | | | 20 (57.1%) | 15 (42.9%) |  |  | 32 (91.4%) | 3 (8.6%) |  |  |
|  | Unilateral mixed | | | 31 (79.5%) | 8 (20.5%) |  |  | 35 (89.7%) | 4 (10.3%) |  |  |
|  | Bilateral mixed | | | 46 (67.7%) | 22 (32.3%) |  |  | 60 (88.2%) | 8 (11.8%) |  |  |
|  | | | | | | | | | | | |
| **GS frequency** | | | |  |  | **0.14**  **(0.02–1.07)** | **0.02** |  |  | 0 | 0.17 |
|  | **Daily** | | | **88 (65.7%)** | **46 (34.3%)** |  |  | 119 (88.8%) | 15 (11.2%) |  |  |
|  | Non-daily | | | 14 (93.3%) | 1 (6.7%) |  |  | 15 (100%) | 0 |  |  |
|  | | | | | | | | | | | |
| Non-GS (+) | | | | 85 (69.1%) | 38 (30.9%) | 0.89  (0.37–2.17) | 0.81 | 110 (89.4%) | 13 (10.6%) | 1.48  (0.31–6.97) | 0.62 |
| Non-GS (-) | | | | 18 (66.7%) | 9 (33.3%) |  |  | 25 (92.6%) | 2 (7.4%) |  |  |
| Non-GS frequency | | | |  |  | 0.60  (0.27–1.31) | 0.20 |  |  | 0.45  (0.14–1.43) | 0.17 |
|  | Daily | | | 27 (61.4%) | 17 (38.6%) |  |  | 37 (84.1%) | 7 (15.9%) |  |  |
|  | Non-daily | | | 56 (72.7%) | 21 (27.3%) |  |  | 71 (92.2%) | 6 (7.8%) |  |  |
|  | | | | | | | | | | | |
| BD | | (+) | | 55 (67.9%) | 26 (32.1%) | 1.08  (0.54–2.16) | 0.83 | 70 (86.4%) | 11 (13.6%) | 2.55  (0.78–7.03) | 0.11 |
|  |  | (–) | | 48 (67.9%) | 21 (30.4%) |  |  | 65 (94.2%) | 4 (5.8%) |  |  |
| ID | | (+) | | 55 (75.3%) | 18 (24.7%) | 0.54  (0.27–1.10) | 0.09 | 68 (93.1%) | 5 (6.9%) | 0.49  (0.16–1.52) | 0.21 |
|  |  | (–) | | 48 (62.3%) | 29 (37.6%) |  |  | 67 (87.0%) | 10 (13.0%) |  |  |
| PP | | | (+) | 38 (77.6%) | 11 (22.4%) | 0.52  (0.24–1.15) | 0.10 | 45 (91.8%) | 4 (8.2%) | 0.73  (0.22–2.41) | 0.60 |
|  |  |  | (–) | 65 (64.4%) | 36 (35.6%) |  |  | 90 (89.1%) | 11 (10.9%) |  |  |
|  | | | | | | | | | | | |
| **Genetic syndrome** | | | **(+)** | **4 (36.4%)** | **7 (63.6%)** | **4.33**  **(1.20–15.61)** | **0.02** | **7 (63.6%)** | **4 (36.4%)** | **6.65**  **(1.68–26.28)** | **0.003** |
|  |  |  | (–) | 99 (71.2%) | 40 (28.8%) |  |  | 128 (92.1%) | 11 (7.9%) |  |  |
|  | | | | | | | | | | | |
| Previous treatment | | | (+) | 30 (73.2%) | 11 (26.8%) | 0.74  (0.34–1.65) | 0.47 | 38 (92.7%) | 3 (7.3%) | 0.64  (0.17–2.39) | 0.50 |
|  |  |  | (–) | 73 (67.0%) | 36 (33.0%) |  |  | 97 (89.0%) | 12 (11.0%) |  |  |
| Open surgery | | | (+) | 18 (81.8%) | 4 (18.2%) | 0.44  (0.14–1.38) | 0.15 | 21 (95.4%) | 1 (4.6%) | 0.39  (0.05–3.11) | 0.36 |
|  |  |  | (–) | 85 (66.4%) | 43 (33.6%) |  |  | 114 (89.1%) | 14 (10.9%) |  |  |
| Endoscopic surgery | | | (+) | 2 (66.7%) | 1 (33.3%) | 1.10  (0.10–12.42) | 0.94 | 3 (100%) | 0 | 0 | 0.56 |
|  |  |  | (–) | 73 (67.0%) | 36 (33.0%) |  |  | 132 (89.8%) | 15 (10.2%) |  |  |
| GKS | | | (+) | 13 (59.1%) | 9 (40.9%) | 1.64  (0.65–4.16) | 0.30 | 20 (90.9%) | 2 (9.1%) | 0.89  (0.19–4.22) | 0.88 |
|  |  |  | (–) | 90 (70.3%) | 38 (29.7%) |  |  | 115 (89.8%) | 13 (10.2%) |  |  |
| Multiple treatment | | | (+) | 5 (62.5%) | 3 (37.5%) | 1.34  (0.31–5.84) | 0.70 | 8 (100%) | 0 | 0 | 0.33 |
|  |  |  | (–) | 98 (69.0%) | 44 (31.0%) |  |  | 127 (89.4%) | 15 (10.6%) |  |  |
|  | | | | | | | | | | | |
| Burr holes  (median, IQR) | | | | 1–3  (2, 1–2) | 1–3  (2, 1–2) |  |  | 1–3  (2, 1–2) | 1–3  (2, 1–2) |  |  |
|  | ≥2 burr holes | | | 53 (67.1%) | 26 (32.9%) | 1.17  (0.58–2.34) | 0.66 | 72 (91.1%) | 7 (8.9%) | 0.77  (0.26–2.23) | 0.62 |
|  | 1 burr hole | | | 50 (70.4%) | 21 (29.6%) |  |  | 63 (88.7%) | 8 (11.3%) |  |  |
| Trajectories  (median, IQR) | | | | 1–11  (4, 3–6) | 1–9  (4, 3–6) |  |  | 1–11  (4, 3–6) | 1–9  (4, 3–7) | 2.39 |  |
|  | >3 trajectories | | | 68 (68.7%) | 31 (31.3%) | 1.00  (0.48–2.07) | 0.99 | 89 (89.9%) | 10 (10.1%) | 1.03  (0.33–3.20) | 0.95 |
|  | ≤3 trajectories | | | 35 (68.6%) | 16 (31.4%) |  |  | 46 (90.2%) | 5 (9.8%) |  |  |
| Coagulations  (median, IQR) | | | | 1–36  (8, 5–14) | 1–36  (8, 5–14) |  |  | 1–36  (8, 5–13) | 3–32  (9, 5–20) |  |  |
|  | >4 coagulations | | | 81 (69.2%) | 36 (30.8%) | 0.89  (0.39–2.03) | 0.78 | 105 (89.7%) | 12 (10.3%) | 1.14  (0.30–4.32) | 0.84 |
|  | ≤4 coagulations | | | 22 (66.7%) | 11 (33.3%) |  |  | 30 (90.9%) | 3 (9.1%) |  |  |

BD, behavioral disorder; CI, confidence interval; GKS, gamma knife radiosurgery; GS, gelastic seizure; HH, hypothalamic hamartoma; ID, intellectual disability; IQR, interquartile range; Non-GS, other types of seizure; OR, odds ratio; PP, precocious puberty; SRT, stereotactic radiofrequency thermocoagulation
