# Supplementary material for: Repeat stereotactic radiofrequency thermocoagulation in patients with hypothalamic hamartoma and seizure recurrence
Source: Epilepsia Open. 2020 Jan 18;5(1):107–20. doi: 10.1002/epi4.12378 (PMC7049799; doi:10.1002/epi4.12378)
Supplement: Supplementary file 3 [file EPI4-5-107-s003.docx]

Table S2. Univariate analyses of clinical factors for non-GS outcomes

|  | | | | Non-GS outcomes after first-SRT | | | | Final non-GS outcomes | | | |
| --- | --- | --- | --- | --- | --- | --- | --- | --- | --- | --- | --- |
|  | | | | Non-GS free | Non-GS recurrence | OR (95% CI) | *P* value | Non-GS free | Non-GS residual | OR (95% CI) | *P* value |
| Number | | | | 90 | 32 |  |  | 90 | 32 |  |  |
|  | | | | | | | | | | | |
| Sex | Male | | | 54 (74.0%) | 19 (26.0%) | 0.97  (0.43–2.22) | 0.95 | 54 (74.0%) | 19 (26.0%) | 0.97  (0.43–2.22) | 0.95 |
|  | Female | | | 36 (73.5%) | 13 (26.5%) |  |  | 36 (73.5%) | 13 (26.5%) |  |  |
| Age at first-SRT, y  (median, IQR) | | | | 1.9­–50  (8.5, 5–16) | 1.7–45  (14, 6.25–28.75) |  |  | 1.8–50  (8.5, 5–16) | 1.7–45  (14, 6.25–28.75) |  |  |
|  | ≤ 8y at first-SRT | | | 45 (81.8%) | 10 (18.2%) | 2.2  (0.94–5.17) | 0.07 | 45 (81.8%) | 10 (18.2%) | 2.2  (0.94–5.17) | 0.07 |
|  | > 8y at first-SRT | | | 45 (67.2%) | 22 (32.8%) |  |  | 45 (67.2%) | 22 (32.8%) |  |  |
| Age at GS onset, y  (median, IQR) | | | | 0–11  (1, 0–3) | 0–6  (0.55, 0–1) |  |  | 0–11  (1, 0–3) | 0–6  (0.55, 0–1) |  |  |
|  | ≤ 1y at GS onset | | | 55 (68.8%) | 25 (31.2%) | 2.27  (0.89–5.81) | 0.08 | 55 (68.8%) | 25 (31.2%) | 2.27  (0.89–5.81) | 0.08 |
|  | > 1y at GS onset | | | 35 (83.3%) | 7 (16.7%) |  |  | 35 (83.3%) | 7 (16.7%) |  |  |
| Duration of GS, y  (median, IQR) | | | | 1–45  (6, 3.375–13) | 1.7–42  (14, 4.25–27.75) |  |  | 1–45  (6, 3.375–13) | 1.7–42  (14, 4.25–27.75) |  |  |
|  | ≤ 6y of GS duration | | | 46 (83.6%) | 9 (16.4%) | **2.67**  **(1.11–6.41)** | **0.02** | 46 (83.6%) | 9 (16.4%) | **2.67**  **(1.11–6.41)** | **0.02** |
|  | **> 6y of GS duration** | | | **44 (65.7%)** | **23 (34.3%)** |  |  | **44 (65.7%)** | **23 (34.3%)** |  |  |
| Age at non-GS onset, y  (median, IQR) | | | | 0–28  (3.75, 1.775–9) | 0–15  (4, 0.5–8) |  |  | 0–28  (3.75, 1.775–9) | 0–15  (4, 0.85–8) |  |  |
|  | ≤ 4y at non-GS onset | | | 49 (74.2%) | 17 (25.8%) | 1.05  (0.47–2.37) | 0.90 | 49 (74.2%) | 17 (25.8%) | 1.05  (0.47–2.37) | 0.90 |
|  | > 4y at non-GS onset | | | 41 (73.2%) | 15 (26.8%) |  |  | 41 (73.2%) | 15 (26.8%) |  |  |
| Duration of non-GS, y  (median, IQR) | | | | 0–45  (3.75, 1–9) | 0–39  (11.1, 3.75–18.975) |  |  | 0–45  (3.75, 1–9) | 0–39  (11.1, 3.75–18.975) |  |  |
|  | ≤ 4y of non-GS duration | | | 50 (83.3%) | 10 (16.7%) | **2.75**  **(1.17–6.47)** | **0.02** | 50 (83.3%) | 10 (16.7%) | **2.75**  **(1.17–6.47)** | **0.02** |
|  | **> 4y of non-GS duration** | | | **40 (64.5%)** | **22 (35.5%)** |  |  | **40 (64.5%)** | **22 (35.5%)** |  |  |
|  | | | | | | | | | | | |
| Maximum diameter of HH, mm | | | | 5–50  (15, 10–21.25) | 9–53  (14.5, 11–24.25) |  |  | 5–50  (15, 10–22) | 9–53  (14.5, 11–21.5) |  |  |
|  | ≤ 15mm | | | 48 (72.7%) | 18 (27.3%) | 0.89  (0.39–2.00) | 0.78 | 48 (72.7%) | 18 (27.3%) | 0.89  (0.39–2.00) | 0.78 |
|  | > 15mm | | | 42 (75.0%) | 14 (25.0%) |  |  | 42 (75.0%) | 14 (25.0%) |  |  |
| HH subtypes | | | |  |  |  | 0.87 |  |  |  | 0.82 |
|  | Parahypothalamic | | | 4 (66.7%) | 2 (33.3%) |  |  | 4 (66.7%) | 2 (33.3%) |  |  |
|  | Intrahypothalamic | | | 22 (78.6%) | 6 (21.4%) |  |  | 22 (78.6%) | 6 (21.4%) |  |  |
|  | Unilateral mixed | | | 27 (75.0%) | 9 (25.0%) |  |  | 27 (75.0%) | 9 (25.0%) |  |  |
|  | Bilateral mixed | | | 37 (71.2%) | 15 (28.9%) |  |  | 37 (69.8%) | 16 (30.2%) |  |  |
|  | | | | | | | | | | | |
| GS frequency | | | |  |  | 0.93  (0.24–3.68) | 0.92 |  |  | 0.93  (0.24–3.68) | 0.92 |
|  | Daily | | | 81 (73.6%) | 29 (26.4%) |  |  | 81 (73.6%) | 29 (26.4%) |  |  |
|  | Non-daily | | | 9 (75.0%) | 3 (25.0%) |  |  | 9 (75.0%) | 3 (25.0%) |  |  |
| **Non-GS frequency** | | | |  |  | **0.17**  **(0.07–0.41)** | **<0.001** |  |  | **0.21**  **(0.09–0.49)** | **<0.001** |
|  | **Daily** | | | **22 (51.2%)** | **21 (48.8%)** |  |  | **23 (53.5%)** | **20 (46.5%)** |  |  |
|  | Non-daily | | | 68 (86.1%) | 11 (13.9%) |  |  | 67 (84.8%) | 12 (15.2%) |  |  |
|  | | | | | | | | | | | |
| CPS | | (+) | | 58 (76.3%) | 18 (23.7%) | 0.71  (0.31–1.61) | 0.41 | 58 (76.3%) | 18 (23.7%) | 0.71  (0.31–1.61) | 0.41 |
|  |  | (–) | | 32 (69.6%) | 14 (30.4%) |  |  | 32 (69.6%) | 14 (30.4%) |  |  |
| **TS** | | **(+)** | | **27 (54.0%)** | **23 (46.0%)** | **5.96**  **(2.44–14.56)** | **<0.001** | **27 (54.0%)** | **23 (46.0%)** | **5.96**  **(2.44–14.56)** | **<0.001** |
|  |  | (–) | | 63 (87.5%) | 9 (12.5%) |  |  | 63 (87.5%) | 9 (12.5%) |  |  |
| GTCS | | (+) | | 43 (70.5%) | 18 (29.5%) | 1.41  (0.62–3.16) | 0.41 | 42 (68.9%) | 19 (31.1%) | 1.67  (0.74–3.79) | 0.21 |
|  |  | (–) | | 47 (77.1%) | 14 (22.9%) |  |  | 48 (78.7%) | 13 (21.3%) |  |  |
| **AS** | | **(+)** | | **7 (46.7%)** | **8 (53.3%)** | **3.95**  **(1.30–12.01)** | **0.01** | **7 (46.7%)** | **8 (53.3%)** | **3.95**  **(1.30–12.01)** | **0.01** |
|  |  | (–) | | 83 (77.6%) | 24 (22.4%) |  |  | 83 (77.6%) | 24 (22.4%) |  |  |
| MS | | (+) | | 2 (40.0%) | 3 (60.0%) | 4.55  (0.72–28.60) | 0.08 | 2 (40.0%) | 3 (60.0%) | 4.55  (0.72–28.60) | 0.08 |
|  |  | (–) | | 88 (75.2%) | 29 (24.8%) |  |  | 88 (75.2%) | 29 (24.8%) |  |  |
| ES | | (+) | | 3 (66.0%) | 2 (40.0%) | 1.93  (0.31–12.13) | 0.47 | 3 (66.0%) | 2 (40.0%) | 1.93  (0.31–12.13) | 0.47 |
|  |  | (–) | | 87 (74.4%) | 30 (25.6%) |  |  | 87 (74.4%) | 30 (25.6%) |  |  |
|  | | | | | | | | | | | |
| BD | | (+) | | 48 (72.7%) | 18 (27.3%) | 1.13  (0.50–2.53) | 0.78 | 47 (71.2%) | 19 (28.9%) | 1.34  (0.59–3.03) | 0.49 |
|  |  | (–) | | 42 (75.0%) | 14 (25.0%) |  |  | 43 (76.8%) | 13 (23.2%) |  |  |
| **ID** | | **(+)** | | **35 (55.6%)** | **28 (44.4%)** | **11.00**  **(3.55–34.06)** | **<0.001** | **36 (57.1%)** | **27 (42.9%)** | **8.10**  **(2.85–23.00)** | **<0.001** |
|  |  | (–) | | 55 (93.2%) | 4 (6.8%) |  |  | 54 (91.5%) | 5 (8.5%) |  |  |
| PP | | | (+) | 27 (71.1%) | 11 (28.9%) | 1.22  (0.52–2.88) | 0.65 | 27 (71.1%) | 11 (28.9%) | 1.22  (0.52–2.88) | 0.65 |
|  |  |  | (–) | 63 (75.0%) | 21 (25.0%) |  |  | 63 (75.0%) | 21 (25.0%) |  |  |
|  | | | | | | | | | | | |
| **Genetic syndrome** | | | **(+)** | **2 (28.6%)** | **5 (71.4%)** | **8.15**  **(1.50–44.41)** | **0.005** | **1 (14.3%)** | **6 (85.7%)** | **20.54**  **(2.36–178.38)** | **<0.001** |
|  |  |  | (–) | 88 (76.5%) | 27 (23.5%) |  |  | 89 (77.4%) | 26 (22.6%) |  |  |
|  | | | | | | | | | | | |
| Previous treatment | | | (+) | 23 (63.9%) | 13 (36.1%) | 1.99  (0.85–4.66) | 0.11 | 23 (63.9%) | 13 (36.1%) | 1.99  (0.85–4.66) | 0.11 |
|  |  |  | (–) | 67 (77.9%) | 19 (22.1%) |  |  | 67 (77.9%) | 19 (22.1%) |  |  |
| Open surgery | | | (+) | 14 (66.7%) | 7 (33.3%) | 1.52  (0.55–4.19) | 0.42 | 14 (66.7%) | 7 (33.3%) | 1.52  (0.55–4.19) | 0.42 |
|  |  |  | (–) | 76 (75.3%) | 25 (24.7%) |  |  | 76 (75.3%) | 25 (24.7%) |  |  |
| Endoscopic surgery | | | (+) | 1 (33.3%) | 2 (66.7%) | 5.93  (0.52–67.79) | 0.11 | 1 (33.3%) | 2 (66.7%) | 5.93  (0.52–67.79) | 0.11 |
|  |  |  | (–) | 89 (74.8%) | 30 (25.2%) |  |  | 89 (74.8%) | 30 (25.2%) |  |  |
| **GKS** | | | **(+)** | **9 (50.0%)** | **9 (50.0%)** | **3.52**  **(1.25–9.90)** | **0.01** | **9 (50.0%)** | **9 (50.0%)** | **3.52**  **(1.25–9.90)** | **0.01** |
|  |  |  | (–) | 81 (77.9%) | 23 (22.1%) |  |  | 81 (77.9%) | 23 (22.1%) |  |  |
| **Multiple treatment** | | | **(+)** | **1 (12.5%)** | **7 (87.5%)** | **24.92**  **(2.93–212.18)** | **<0.001** | **1 (12.5%)** | **7 (87.5%)** | **24.92**  **(2.93–212.18)** | **<0.001** |
|  |  |  | (–) | 89 (78.1%) | 25 (21.9%) |  |  | 89 (78.1%) | 25 (21.9%) |  |  |
|  | | | | | | | | | | | |
| Burr holes  (median, IQR) | | | | 1–3  (1, 1–2) | 1–3  (2, 1–2) |  |  | 1–3  (1.5, 1–2) | 1–3  (2, 1–2) |  |  |
|  | ≥2 burr holes | | | 43 (68.3%) | 20 (31.7%) | 1.61  (0.71–3.62) | 0.25 | 45 (70.3%) | 19 (29.7%) | 1.36  (0.61–3.03) | 0.46 |
|  | 1 burr hole | | | 47 (79.7%) | 12 (20.3%) |  |  | 45 (76.3%) | 14 (23.7%) |  |  |
| Trajectories  (median, IQR) | | | | 1–11  (4, 3–5) | 1–9  (4, 3–6.75) |  |  | 1–11  (4, 3–5.25) | 1–9  (4, 3–6.75) |  |  |
|  | >3 trajectories | | | 57 (71.2%) | 23 (28.8%) | 1.33  (0.57–3.14) | 0.51 | 57 (71.2%) | 23 (28.8%) | 1.33  (0.57–3.14) | 0.51 |
|  | ≤3 trajectories | | | 33 (76.7%) | 10 (23.3%) |  |  | 33 (76.7%) | 10 (23.3%) |  |  |
| Coagulations  (median, IQR) | | | | 1–36  (8, 5–12.25) | 1–32  (8, 6–16.25) |  |  | 1–36  (8, 5–12.25) | 1–32  (8, 6–16.25) |  |  |
|  | >4 coagulations | | | 69 (71.9%) | 27 (28.1%) | 1.37  (0.50–3.76) | 0.54 | 69 (71.9%) | 27 (28.1%) | 1.37  (0.50–3.76) | 0.54 |
|  | ≤4 coagulations | | | 21 (77.8%) | 6 (22.2%) |  |  | 21 (77.8%) | 6 (22.2%) |  |  |

AS, atonic seizure; BD, behavioral disorder; CI, confidence interval; CPS, complex partial seizure; ES, epileptic spasms; GKS, gamma knife radiosurgery; GS, gelastic seizure; GTCS, generalized tonic-clonic seizure; HH, hypothalamic hamartoma; ID, intellectual disability; IQR, interquartile range; MS, myoclonic seizure; Non-GS, other types of seizure; OR, odds ratio; PP, precocious puberty; SRT, stereotactic radiofrequency thermocoagulation; TS, tonic seizure
